# NVVC/NHJ Durrer prizes 2013

**DOI:** 10.1007/s12471-014-0548-6

**Published:** 2014-03-26

**Authors:** E. E. van der Wall, V. A. W. M. Umans

**Affiliations:** 1 Interuniversity Cardiology Institute of the Netherlands (ICIN)–Netherlands Heart Institute, Catherijnesingel 52, P.O. Box 19258, 3501 DG Utrecht, the Netherlands; 2 Department of Cardiology, Medical Center Alkmaar, Wilhelminalaan 12, 1815 JD Alkmaar, the Netherlands

**Keywords:** Durrer prize, Congress NVVC, Netherlands Heart Journal

## Abstract

At the annual Spring Congress of the NVVC, the Durrer prizes were awarded to the authors of two of the best original/review articles published in the year 2013, one paper being more basically oriented and one paper being more clinically oriented. This annual tradition has existed since the year 2006.

For the eighth time in a row, the Netherlands Heart Journal (NHJ), the official journal of the Netherlands Society of Cardiology (NVVC), awarded the Durrer Prize to the two best NHJ articles published in the year 2013. In 2006, it was thought appropriate by the NVVC Board to set up a special publication prize in order to stimulate the submission of outstanding scientific articles to NHJ. Two articles per year are selected, one article with a more basically oriented character and one with a mainly clinical focus [1–3].

The NHJ publication prize carries the name of one of the fathers of Dutch Cardiology, Professor Dirk Durrer (1918–1984), Head of the Department of Cardiology in the ‘Wilhelmina Gasthuis’, Amsterdam, who performed pioneering work in the field of electrical activation of the heart in the 1960s and 1970s [4]. Furthermore, Dirk Durrer founded the Interuniversity Institute of the Netherlands (ICIN) in 1972, of which he was director until 1983.

From a total of 140 articles published in NHJ in 2013, we selected as best basically oriented article: *Ebstein’s anomaly may be caused by mutations in the sarcomere protein gene MYH7. Authors: van Engelen K, Postma AV, van de Meerakker JB, Roos-Hesselink JW, Helderman-van den Enden AT, Vliegen HW, Rahman T, Baars MJ, Sels JW, Bauer U, Pickardt T, Sperling SR, Moorman AF, Keavney B, Goodship J, Klaassen S, Mulder BJ. Neth Heart J. 2013;21:113–7. doi:* 10.1007/s12471-011-0141-1. Recent studies have shown an association between Ebstein’s anomaly with left ventricular noncompaction (LVNC) and mutations in the sarcomeric protein gene MYH7, encoding beta-myosin heavy chain. In this article the association of MYH7 mutations with Ebstein’s anomaly and LVNC and its implications for the clinical care of patients and their family members was discussed. This national multicentre study has been cited, among others, by the American Journal of Medical Genetics and Molecular Genetics and Metabolism.

As best clinical article, we selected: *Long-term performance of the St Jude Riata 1580–1582 ICD lead family. Authors: Valk SD, Theuns DA, Jordaens L. Neth Heart J. 2013;21:127–34. doi:* 10.1007/s12471-012-0341-3. In 374 patients of the Erasmus Medical Centre, Rotterdam, a high incidence of insulation defects associated with conductor externalisation in the Riata ICD lead family was observed between July 2003 and December 2007. This type of insulation failure can lead to failure of therapy delivery. The article has been cited, among others, by the Journal of Interventional Cardiac Electrophysiology, Pace-Pacing and Clinical Electrophysiology, and Heart Rhythm.

At the annual spring meeting of the NVVC, held at the RAI Congress Centre on Friday 4 April 2014, the first authors of both articles received an educational grant provided by the NVVC. We would like to congratulate the authors on their awards and thank them for sending their excellent work to NHJ. With the Durrer Prize, we again hope to stimulate young investigators to send their best papers to NHJ.
